# Melioidosis in Trinidad and Tobago

**DOI:** 10.3201/eid2105.141610

**Published:** 2015-05

**Authors:** Catherine Hogan, Amanda Wilmer, Mazen Badawi, Linda Hoang, Michael Chapman, Natasha Press, Kym Antonation, Cindi Corbett, Marc Romney, Melanie Murray

**Affiliations:** University of Sherbrooke, Sherbrooke, Quebec, Canada (C. Hogan);; University of British Columbia, Vancouver, British Columbia, Canada (A. Wilmer, M. Badawi, L. Hoang, M. Chapman, N. Press, M. Romney, M. Murray);; King Abdulaziz University, Jeddah, Saudi Arabia (M. Badawi);; British Columbia Centre for Disease Control Public Health Microbiology and Reference Laboratory, Vancouver (L. Hoang);; St. Paul’s Hospital, Vancouver (N. Press, M. Romney, M. Murray);; National Microbiology Laboratory, Winnipeg, Manitoba, Canada (K. Antonation, C. Corbett);; British Columbia Women’s Hospital, Vancouver (M. Murray)

**Keywords:** *Burkholderia pseudomallei*, melioidosis, epidemiology, Trinidad and Tobago, bacteria

**To the Editor:** Melioidosis refers to infection caused by the facultative intracellular gram-negative bacterium *Burkholderia pseudomallei*. The clinical manifestations of melioidosis span a wide spectrum, from asymptomatic exposure or localized cutaneous infection to septic shock with multiorgan failure. Melioidosis usually occurs in residents of or travelers to disease-endemic areas in northern Australia and Southeast Asia; however, an increasing number of confirmed melioidosis cases are being reported from the Caribbean. We report a case of melioidosis acquired in Trinidad and Tobago.

In February 2014, a 17-year-old male student was admitted to a tertiary care hospital in Vancouver, British Columbia, Canada, with catecholaminergic polymorphic ventricular tachycardia and electrical storm. He had a 9-month history of dry cough that was unresponsive to multiple and prolonged courses of treatment for community-acquired pneumonia. During the 6 months before his admission, the patient had hemoptysis and radiologic evidence of pneumonia that were treated with courses of cephalosporins without resolution of symptoms. Bronchoscopy and culture of lavage samples had revealed infection with *Staphylococcus aureus* and an organism most closely related to *Actinomyces graevenitzii* *.*

The patient had no history indicative of risk factors for recurrent sinusitis or pneumonia (e.g., cystic fibrosis, chronic granulatomous disease, Job syndrome), and no risk factors for tuberculosis or infection with dimorphic fungi. He was up to date on his vaccinations and had no pets. He was born in Jamaica, had moved to Canada at age 4, and had not traveled anywhere other than Trinidad and Tobago, Canada, and England. He had traveled to visit family in Trinidad for 2 months during the rainy season in 2012, at which time he also visited Tobago.

On day 5 of hospital admission, the patient became febrile (39.6°C), and an infectious diseases specialist was consulted. Examination revealed that the patient was clinically stable but emaciated at 45 kg. His oxygen saturation while breathing room air was 98%. Physical examination, including cardiorespiratory examination, was unremarkable. Laboratory results showed a normal hemoglobin concentration of 133 g/L; elevated leukocyte count of 22.8 × 10^9^ cells/L; neutrophils 19.4 × 10^9^ cells/L; normal platelet count of 295 × 10^9^/L; and normal creatinine of 54 μmol/L. Test results for HIV-1 and blood cultures were negative. Computed tomography scan showed dilated bronchi and dense consolidation of the right and left lower lobes. Piperacillin/tazobactam was started for presumed hospital-acquired pneumonia.

The patient underwent diagnostic bronchoscopy with bronchoalveolar lavage. Gram staining of specimens showed occasional gram-negative bacilli, and aerobic cultures grew gram-negative bacilli. Further testing with the Vitek 2 (bioMérieux, Laval, Quebec, Canada) (96%) and RapID NF (Oxoid, Nepean, Ontario, Canada) (99.9%) systems identified *B. pseudomallei*, but matrix-assisted laser desorption/ionization time-of-flight mass spectrometry (Vitek MS, bioMérieux) did not. Phenotypic confirmation was performed at the provincial public health and reference laboratory. Antimicrobial drug susceptibility testing performed by broth microdilution according to Clinical and Laboratory Standards Institute recommendations (*1*) and by Etest (bioMérieux) showed susceptibility to amoxicillin/clavulanic acid, ceftazidime, imipenem, doxycycline, and trimethoprim/sulfamethoxazole. The patient’s condition improved after 2 weeks of intravenous meropenem, and antimicrobial therapy was changed to oral trimethoprim/sulfamethoxazole.

The *B. pseudomallei* isolate was sent to the Public Health Agency of Canada’s National Microbiology Laboratory for molecular typing. Query of 7 standard multilocus sequence typing loci (http://bpseudomallei.mlst.net/) identified the isolate as a novel multilocus sequence type. The sequence type (1,1,2,1,5,6,1) closely resembled that of *B. pseudomallei* previously isolated from the Caribbean (*2*).

Although melioidosis was first described in the Caribbean in 1947 (*3*), most case reports of the disease in the area are from the past 2 decades. This case report suggests progression of the range of melioidosis to include Trinidad and Tobago. A recent study documented the presence of *B. pseudomallei* in soil samples and high seroprevalence rates among contacts of persons with melioidosis in Puerto Rico (*4*)*.* If examined, this pattern of regional melioidosis endemicity may also be found on other Caribbean islands.

Increased clinical awareness of and improved surveillance for *B. pseudomallei* infection may partly explain emergence. Nonetheless, underascertainment probably occurs in rural areas with limited access to advanced diagnostic support and in urban areas when *B. pseudomallei* infection is not suspected because of lack of travel to classic disease-endemic areas. Because *B. pseudomallei* is a Biosafety Level 3 agent, when infectious disease specialists consider melioidosis in their differential diagnoses, they should alert the microbiology laboratory to confirm species identification and ensure that staff use proper biosafety measures.

A total of 19 cases of melioidosis acquired in the Caribbean have been reported (Table). Nine of these were travel related, suggesting that melioidoisis may be emerging as a travel health issue. Travelers with known risk factors for melioidosis, such as diabetes mellitus and chronic lung disease, should be informed of their increased infection risk. Physicians should include *B. pseudomallei* in the differential diagnosis of travelers with pneumonia or sepsis who are returning from the Caribbean, particularly when they have a history of travel during the rainy season, soil-contaminated wounds, or known risk factors for melioidosis.

**Table:** Published case reports of melioidosis from the Caribbean*

Ref.	Site of origin (year)	Age, y/sex	Type of exposure	Concurrent condition	Clinical manifestation	Diagnostic method	Treatment (duration)	Outcome
(*3*)	Panama (1947)	31/M	Fall on buttock, TR	Polio, spinal meningitis	Buttock abscess	Abscess culture	Sulfathiazole, sulfapyridine, streptomycin, penicillin	Survived
(*5*)	Panama (1948)	25/F	UNK, TR	None	Retroperitoneal abscess, sepsis	Abscess culture	Penicillin, streptomycin	Died
(*5*)	Panama (1960)	20/M	UNK	None	Acute septic arthritis	Synovial fluid culture	Chloramphenicol, novobiocin, sulfisoxazole	Survived
(*5*)	Puerto Rico (1982)	62/F	UNK	Diabetes, SLE, cirrhosis	Septic meningitis	Blood and CSF culture	Penicillin, chloramphenicol, moxalactam, amikacin	Died
(*5*)	Mexico (1986)	72/M	UNK	None	Pneumonia, splenic abscess	Blood and sputum culture	Cefoxitin, gentamicin	Died
(*5*)	Martinique (1995)	66/M	UNK, resident	Diabetes	Sepsis	Blood and urine culture	IV ceftazidime, then oral TMP/SMX and doxycycline (2 mo)	Survived
(*5*)	Guadeloupe (1997)	4/M	UNK, TR	None	Pneumonia, pleural effusion, peritonitis	Pleural fluid culture	IV ceftazidime and TMP/SMX(1 mo), then oral TMP/SMX (6 mo)	Survived
(*5*)	Puerto Rico (1997)	11/M	UNK, resident	X-linked CGD	Mediastinitis, lymphadenitis	Supraclavicular and hilar biopsy culture	Imipenem and doxycycline (6 weeks), oral cefixime and doxycycline (3 wk)	Died†
(*5*)	El Salvador (2001)	UNK	UNK, TR	UNK	Cerebral abscess	UNK	UNK	Survived
(*4*)	Puerto Rico (2003)	55/F	Flood water, resident	Diabetes	Pneumonia, septic shock	Blood and sputum culture	Imipenum, amikacin, azithromycin	Died
(*6*)	British Virgin Islands (2006)	17/M	UNK, resident	CF	Pneumonia	Sputum culture, PCR	UNK	Survived
(*7*)	Aruba (2009)	7/F	UNK, TR	CF	Pneumonia	Oropharyngeal and induced sputum culture	Imipenem and ceftazidime (14 d), then inhaled meropenem (28 d) and long-term oral TMP/SMX	Survived
(*4*)	Puerto Rico (2009)	88/M	Ditch digging, resident	CAD, PVD	Pneumonia	MLST	Doxycycline (20 wk), oral TMP/SMX	Survived
(*8*)	Guadeloupe (2010)	15/F	UNK, TR	Asthma, dengue fever	Adenopathy, tumefaction	Tumefaction culture PCR	IV ceftazidime (10 d), then oral TMP/SMX (12 wk)	Survived
(*9*)	Martinique (2010)	35/M	UNK, TR	None	Diarrhea, pneumonia	Blood culture; PCR	Imipenem, G-CSF	Died
(*10*)	Aruba (2012)	46/F	Water exposure, TR	None	Breast abscesses	Abscess culture	Meropenem (14 d), then oral TMP/SMX (12 wk)	Survived
(*4*)	Puerto Rico (2010)	38/M	Landscaping, resident	None	Pneumonia, hepatitis, myocarditis, septic shock	Immunohistochemistry with polyclonal Ab; PCR	None	Died
(*4*)	Puerto Rico (2012)	60/M	Agricultural work, resident	Diabetes	Diabetic ketoacidosis	Blood culture; MLST	Amoxicillin/cloxacillin (10 d), then oral TMP/SMX (12 weeks)	Survived
This study	Trinidad and Tobago (2014)	17/M	Rainy season, TR	CPVT	Chronic pneumonia	BAL culture; MLST	Meropenem (2 weeks), then oral TMP/SMX (8 mo to date)	Survived

*Ab, antibody; BAL, bronchoalveolar lavage; CAD, coronary artery disease; CGD, chronic granulomatous disease; CPVT, catecholaminergic polymorphic ventricular tachycardia; CSF, cerebrospinal fluid; G-CSF, granulocyte colony-stimulating factor; IV, intravenous; MLST, multilocus sequence typing; PVD, peripheral vascular disease; Ref., reference; SLE, systemic lupus erythematosus; TMP/SMX, trimethoprim/sulfamethoxazole; TR, travel related; UNK, unknown. †Cause of death unknown.
